# Flavodiiron proteins in *Physcomitrium patens*: navigating the edge between photoprotection and efficiency

**DOI:** 10.1111/tpj.70052

**Published:** 2025-02-24

**Authors:** Eleonora Traverso, Claudia Beraldo, Marco Armellin, Alessandro Alboresi, Tomas Morosinotto

**Affiliations:** ^1^ Department of Biology University of Padova Via Ugo Bassi 58/B Padova 35131 Italy; ^2^ National Biodiversity Future Center Università degli Studi di Palermo Palermo Italy

**Keywords:** photosynthesis, photoprotection, alternative electron transport, photosynthetic electron transport regulation, flavodiiron proteins, moss

## Abstract

Sunlight is the primary energy source for photosynthetic organisms, driving electron transport that supports the synthesis of ATP and NADPH. In dynamic environmental conditions, photosynthetic electron transport requires continuous modulation to prevent over‐reduction and safeguard against potential damage. Flavodiiron proteins (FLV) contribute to photoprotection by accepting electrons downstream of Photosystem I, reducing oxygen to water. FLV were shown to have a seminal role in response to abrupt changes in illumination intensity in various photosynthetic organisms, such as cyanobacteria, green algae, mosses, and gymnosperms but were lost during evolution of angiosperms. In this work, *Physcomitrium patens* plants with strong FLV accumulation, up to 20 times higher than WT, were isolated. Overexpressor plants exhibited faster activation of electron transport but did not gain additional tolerance to light fluctuations, suggesting that the contribution to photoprotection from the FLV was already saturated in WT plants. On the contrary, strong protein overexpression caused a growth penalty under steady low or high light intensity suggesting that FLV overaccumulation can be detrimental, at least in some conditions, opening hypotheses to explain why these proteins were lost during the evolution of angiosperms.

## Significance Statement

Flavodiiron proteins are active in the photoprotection of photosynthetic organisms exposed to light fluctuations. This study identifies some potential drawbacks of FLV overexpression, opening hypotheses for their evolutionary loss in angiosperms.

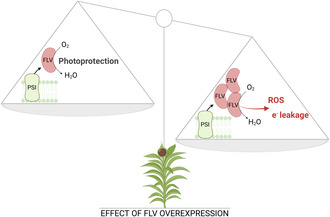

In many photosynthetic organisms, Flavodiiron proteins play an important role in response to light fluctuations. However, they were lost during the evolution of angiosperms. This study highlights the potential drawbacks associated with FLV overexpression, providing hypotheses that might have driven their evolutionary loss in angiosperms despite their role in photoprotection.

## INTRODUCTION

Plants, algae, and cyanobacteria harness sunlight for the synthesis of NADPH and ATP, essential for sustaining cellular metabolism. This process involves the conversion of light energy into chemical energy catalyzed by two pigment‐protein complexes, Photosystem (PS) II and PSI, which mediate the transfer of electrons from water to NADP^+^. The utilization of light energy involves the generation of excited pigment states and multiple electron transport reactions, that carry the risk of formation of undesirable by‐products, such as reactive oxygen species (ROS). This risk becomes particularly significant in highly dynamic environments (Allahverdiyeva et al., 2015; Kulheim et al., 2002).

To thrive in such dynamic conditions, photosynthetic organisms evolved various mechanisms to modulate photosynthetic reactions. These include several alternative electron transport pathways capable of adjusting ATP and NADPH production by either channeling or diverting electrons from the main electron transport chain in response to metabolic and environmental constraints (Alboresi et al., 2019; Burlacot & Peltier, 2023; Shikanai & Yamamoto, 2017). The regulation of photosynthetic electron transport involves, for instance, the modulation of proton accumulation in the lumen that drives ATP biosynthesis but also can inhibit the activity of cytochrome (Cyt) b_6_f, thereby preventing over‐reduction of PSI. Mitochondrial respiratory activity also impacts photosynthetic electron transport by consuming reduced power exported from the chloroplast (Burlacot, 2023). Another regulatory mechanism is cyclic electron flow (CEF) around PSI which directs electrons from PSI back to plastoquinone (PQ) or Cyt b_6_f thus generating a proton gradient for ATP production without concurrent NADPH formation. CEF thus adjusts electron transport under diverse illumination conditions across various plant species (Shikanai & Yamamoto, 2017).

A further contribution to regulation is provided by the water–water cycle, also known as pseudo cyclic electron flow (PCEF), where electrons from PSI are donated back to oxygen, regenerating water. This process also results in the production of ATP without NADPH synthesis. PCEF includes the Mehler reaction, vital for detoxifying ROS originating from PSI (Asada, 2000), and the electron transport catalyzed by flavodiiron proteins (FLV) (Allahverdiyeva et al., 2013) which accept electrons directly from ferredoxin (Sétif et al., 2020). FLV function as electron sinks downstream PSI and provide a transient yet quantitatively significant contribution to electron transport rates (ETR) (Gerotto et al., 2016). When active, FLV establishes an additional transient transport pathway thus preventing PSI over‐reduction and averting potential damage. FLV activity is particularly impactful during abrupt increases in illumination, and indeed, they play a pivotal role in photoprotection and the regulation of photosynthesis under fluctuating light conditions in various organisms including cyanobacteria, green algae, non‐vascular plants, and gymnosperms (Allahverdiyeva et al., 2013, 2015; Bag et al., 2023; Chaux et al., 2017; Gerotto et al., 2016; Ilík et al., 2017).

FLV consist of two subunits, named FLV1/3 in cyanobacteria and the homologous FLVA/B in eukaryotes. In several cyanobacteria, additional homologous subunits (FLV2/4) have been identified (Santana‐Sanchez et al., 2019). FLV assemble into heterocomplexes (Wada et al., 2018), where the absence of any subunit compromises the stability and activity of the entire complex (Allahverdiyeva et al., 2015; Gerotto et al., 2016). FLV identified in photosynthetic organisms belong to a larger class of flavodiiron proteins (FDP) present also in several anaerobic bacteria. FDP protect from oxidative and nitrosative stress by reducing oxygen to water or NO to NO_2_ (Martins et al., 2019; Rodrigues et al., 2006; Romão et al., 2016).

Even though FLV have been shown to play a major role in responding to fluctuating light across various photosynthetic organisms from cyanobacteria to mosses and gymnosperms, they were lost during the evolution of angiosperms (Bag et al., 2023; Ilík et al., 2017). The loss of FLV activity in angiosperms was suggested to be compensated by an enhanced CEF as PSI photoprotective mechanism (Rizzetto et al., 2025; Storti et al., 2019; Yamamoto et al., 2016). Indeed, the moss *Physcomitrium patens* possesses both CEF and FLV, but mutants lacking the latter exhibit an increased cyclic activity thus supporting the hypothesis of a compensatory relationship between these two mechanisms (Gerotto et al., 2016; Storti, Puggioni, et al., 2020). Studies with Arabidopsis, rice, and tobacco plants overexpressing FLV demonstrate improved PSI protection against over‐reduction without negative effects on carbon fixation efficiency (Basso et al., 2022; Suganami et al., 2022; Vicino et al., 2021; Wada et al., 2018). These results show how FLV are functional in angiosperms with no obvious negative effects, making their evolutionary loss harder to explain.

In this study, moss plants were engineered to accumulate very high levels of FLV. FLV overexpression rescued electron transport activity and photosensitivity to light fluctuations of *flv* KO recipient plants, but protein overaccumulation did not provide any additional advantage in photoprotection. Unexpectedly, an increased FLV accumulation is associated with a growth penalty under constant illumination, both low and intense, suggesting that FLV have a small constitutive activity that may become detrimental especially if the protein is over‐accumulated.

## RESULTS

### Isolation of *P. patens* plants with different FLV accumulation levels

Gene targeting by homologous recombination was exploited to generate null mutant plants for both FLVA and FLVB (*flva/b* KO) in *P. patens*. Transformation of *flva* KO plants with the PpFLVB‐targeting construct (Gerotto et al., 2016) yielded several independent lines where the effectiveness of homologous recombination and *PpFLVB* gene disruption was confirmed by RT‐PCR (Figure S1a). FLV electron transport activity impacted the kinetics of chlorophyll fluorescence quenching, that under mild illumination was slower in *flva/b* KO with respect to wild‐type (WT, Figure S1b). The phenotype of double *flva/b KO* was indistinguishable from the one of the single *flva* and *flvb* KO (Figure S1b), confirming previous indications that these two proteins belong to the same functional complex and that the absence of either protein causes the complete loss of FLV activity (Gerotto et al., 2016).

*flva/b KO* line was complemented with a construct expressing both FLVA and FLVB coding sequences separated by a 2A peptide (Figure S2a), a strategy already shown to be effective for these proteins (Yamamoto et al., 2016). This approach was chosen to achieve a similar expression level of the two subunits and thus an optimal stoichiometry in heterologous complexes (Figure S2a; Allahverdiyeva et al., 2015). *flva/b* KO was chosen as the background for the transformation to avoid any possible influence from the native proteins. Transformed plants showing antibiotic resistance were screened using Chl fluorescence imaging, exploiting the different kinetics of fluorescence quenching between WT and *flva/b* KO plants (Figure S2b–g). The fluorescence phenotype typical of WT was rescued in 12 independent lines indicating a possible recovery of FLV activity (Figure S2b–g).

**Figure 1:**
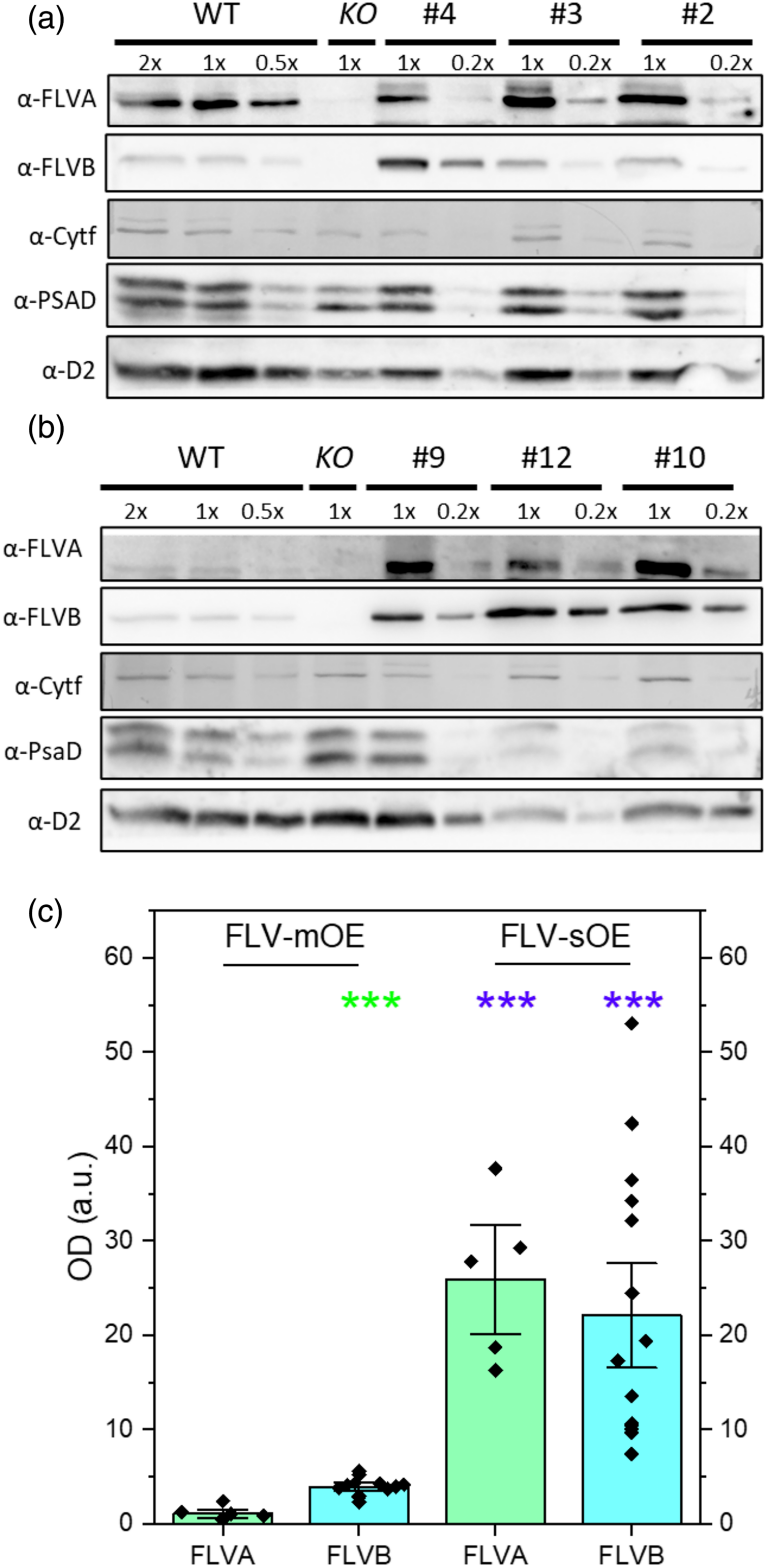
Quantification of FLVA and FLVB protein accumulation. (a, b) Immunoblot analysis of 10‐days‐old plants overexpressing FLV. WT, *flva/b KO* and 6 independent FLV‐OE lines (#2, 3, 4, 9, 10, 12) are shown. Different amounts of protein extract were loaded to avoid antibody signal saturation (1× corresponds to the equivalent of 3 μg of Chl). FLVA and FLVB content was assessed together with PsaD, D2 and Cytf as representative subunits of PSI, PSII, and Cyt b6f respectively. (a) Lines #2, 3, 4 are named FLV‐mOE. (b) Lines #9, 10, 12 are named FLV‐sOE. (c) FLVA (green) and FLVB (cyan) relative protein content were quantified by densitometry of western blotting. In all cases, band intensity was normalized to the signal from WT plants in the same blot. Graph reports data of *n* > 4 biological replicates. FLV‐sOE and FLV‐mOE report data from three independent lines with *n* > 4 each. Asterisks indicate significant differences between WT and the correspondent lines (*t*‐test, ***P < 0.01).

Figure 1(a,b) shows the accumulation levels of both FLVA and FLVB in six independent lines. Three overexpressing lines (# 2, 3, 4, called FLV‐mild Over Expressor, FLV‐mOE) presented an FLVA and FLVB accumulation level 2–3 times higher than WT plants (Figure 1a,c) while three others (#9, 12, 10, called FLV‐strong Over Expressor, FLV‐sOE) had approximately 20 times higher FLV accumulation (Figure 1b,c). No plants with FLV accumulation close to WT level were isolated, likely because of the use of a strong promoter. Lines showing the strongest FLVA accumulation also had high content of FLVB, consistent with their tendency to form heterocomplexes. All overexpressing plants showed no major alterations in the accumulation level of photosynthetic electron transport components (PSI, PSII, Cytb6f, Figure 1a,b; Figure S2h).

### FLV overexpression rescues electron transport activity upon light activation

**Figure 2:**
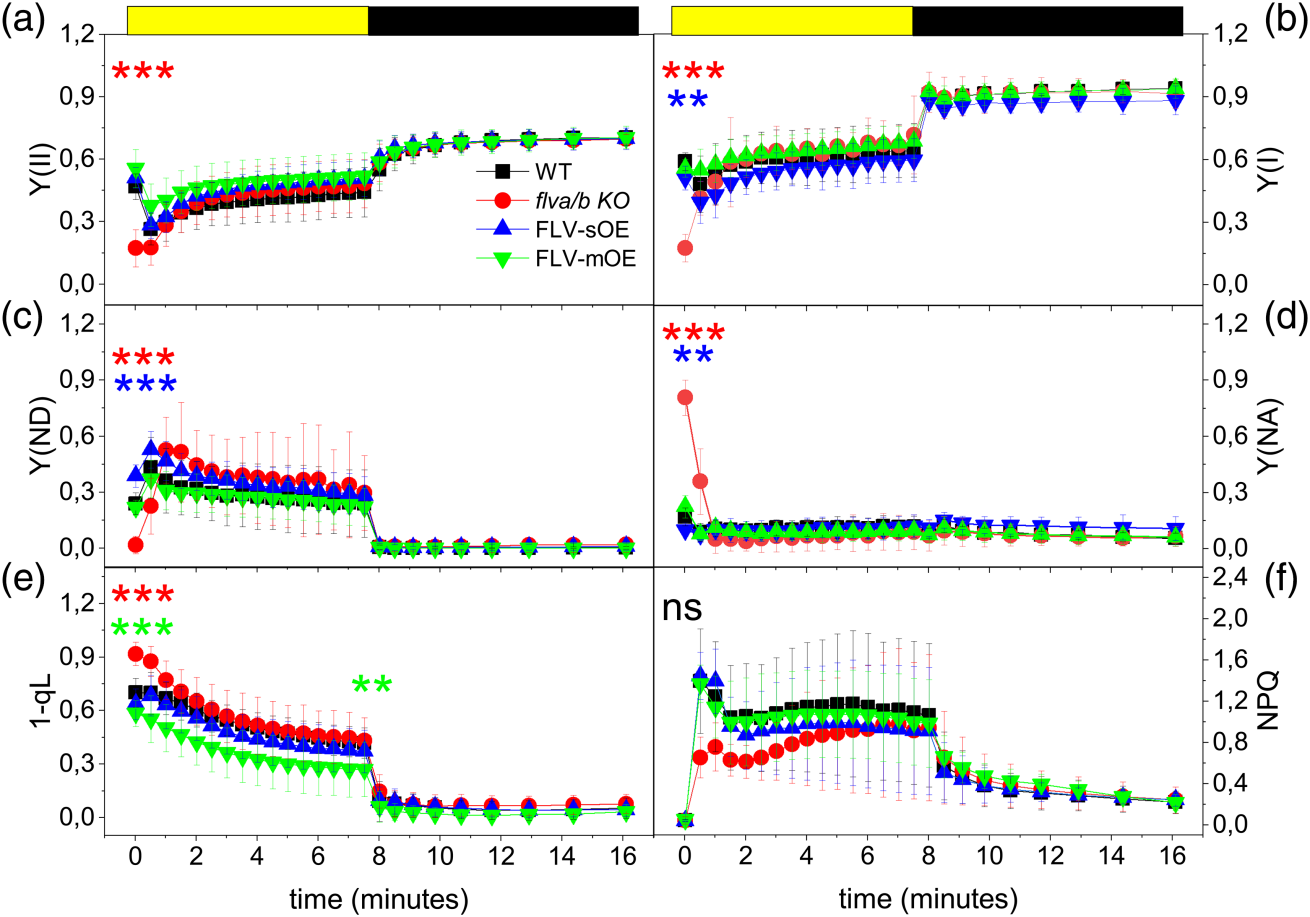
Impact of FLV overexpression on PSII and PSI activity and energy dissipation as heat (NPQ). Dark‐adapted *Physcomitrium patens* plants were exposed for 8 min to 165 μmol photons m^2^ sec^−1^ light followed by 8 min of darkness. (a–f) Graphs report respectively Y(II), Y(I), Y(ND), Y(NA), fraction of PSII closed reaction centers (1‐qL) and NPQ values. WT, *flva/b* KO, FLV‐mOE, and FLV‐sOE lines are shown respectively as black squares, red circles, green triangles, and blue triangles. FLV‐sOE and FLV‐mOE reports averaged data from three independent lines. Statistically significant differences between WT and mutant lines, at the beginning and end of light treatment, are indicated by asterisks with the color code of the correspondent lines (*t*‐test, **P = 0.01; ***P < 0.01).

FLV activity *in vivo* was verified in all complemented lines by measuring PSI and PSII yield [respectively Y(I) and Y(II)] during dark‐to‐light transition. Immediately after the light is switched‐on, Y(I) was lower in *flva/b* KO than in WT (Figure 2b). Upon the dark‐to‐light transition, the PSI activity of WT plants was limited from the donor side, while *flva/b* KO experienced a strong PSI acceptor side limitation (Figure 2c,d; Figure S4). All complemented lines showed a complete rescue of *flva/b* KO phenotype with a high PSI donor side limitation upon illumination (Figure 2c). A deeper focus on the first seconds of light exposure highlighted how FLV‐mOE showed PSI acceptor and donor side limitation levels close to WT ones (Figure S4) while FLV‐sOE plants had an even lower acceptor side limitation and a larger donor side limitation than WT (Figure S4). Interestingly, the difference between FLV‐sOE and WT increased with the light intensity (Figure S3c,d), suggesting that an augmented FLV accumulation provided a slightly larger electron transport capacity downstream of PSI upon a light transition (Figure S3c,d). FLV activity also impacted PSII activity, as shown by the increased fraction of PSII closed reaction centers, 1‐qL, in *flva/b* KO plants (Figure 2a–e). The activation of excess energy dissipation mechanism (NPQ) was instead not altered by FLV accumulation (Figure 2f).

After 2 min of illumination, however, the difference between WT and *flva/b* KO recovered and both Y(I) and Y(II) became indistinguishable between genotypes (Figure 2a,b). Independently from the FLV accumulation levels, overexpressors showed a rescue of *flva/b* KO phenotype and their Y(I) and Y(II) appeared indistinguishable from WT upon dark‐to‐light transitions (Figure 2), even if exposed to strongly saturating illumination (Figure S3a,b).

Considering that FLV activity is particularly impactful during light fluctuations, PSI maximal efficiency (Pm) was monitored before (Pm_i_) and after (Pm_f_) plants exposure to strong light fluctuations for 60 min. *flva/b* KO showed a significant drop in Pm_f_, much larger than WT (Figure S5) that was likely associated with PSI damage. Pm_f_ was rescued to WT levels in all overexpressing lines thus confirming the major role of FLV in fast response to light fluctuations (Figure S5), but also suggesting that a higher FLV accumulation did not yield any additional advantage in this condition.

**Figure 3:**
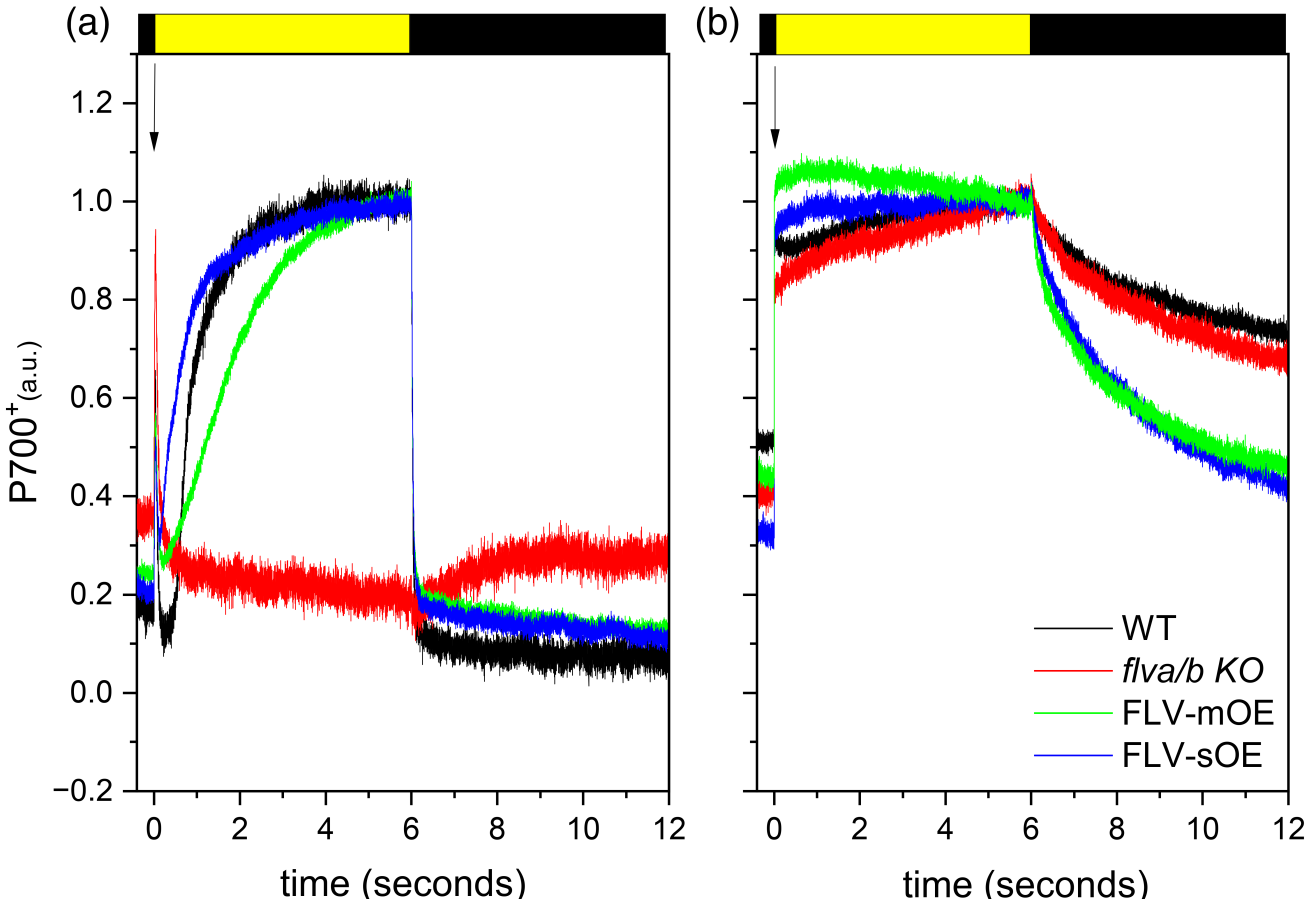
FLV‐OE lines keep P700 oxidized in the dark‐to‐light transition. P700 oxidation state was monitored in *Physcomitrium patens* dark‐adapted plants, not‐treated or treated with DCMU respectively in (a, b), illuminated for 6 sec (20 000 μmol photons m^−2^ sec^−1^) using DUAL‐PAM‐100. WT and *flva/b* KO are shown in black and red respectively while FLV‐mOE and FLV‐sOE are shown in green and blue. Traces were normalized to the maximal oxidation level after the light was switched on (black arrow). FLV‐sOE and FLV‐mOE reports averaged data from three independent lines. All traces are average of *n* > 4 independent experiments.

The impact of FLV accumulation on PSI activity can be more precisely estimated by monitoring PSI redox state and electron transport rate (ETR) upon light activation. The light‐driven P700 redox kinetic has been evaluated on dark‐adapted plants exposed to a short (6 sec) saturating pulse (20 000 μmol photons m^−2^ sec^−1^, Helman et al., 2003; Ilík et al., 2017). All lines showed a transient peak of P700 oxidation (P700^+^) (black arrow in Figure 3a) resulting from PSI charge separation and electrons coming from PSII and plastocyanin as confirmed by the peak absence in DCMU‐treated plants (Figure 3b). In *P. patens flva/b* KO, the P700 reduced pool quickly increased and reaction centers remained reduced for the whole 6 sec of the light treatment (Figure 3a). In WT plants, instead, P700 reaction centers were largely oxidized during the illumination treatment, reaching a maximum within 3 sec (Figure 3a). This different light‐dependent behavior was associated with FLV capacity to maintain P700 pool oxidized during this short light treatment, as previously shown (Ilík et al., 2017; Shimakawa et al., 2018) (Figure 3a). All *P. patens* complemented lines (FLV‐OEs) exhibited a WT‐like P700 redox kinetic, further confirming that they accumulated active forms of the protein. Furthermore, WT and FLV‐OEs showed overlapping maximal P700 oxidation capacity both at the end of the 6 sec saturating light exposure and after repetitive saturating pulses (Figure S6a). However, a clear difference was visible in the first second after the dark‐to‐light transition (Figure S6b). The light‐driven P700 oxidation was faster in FLV‐sOE lines (half of the maximal P700 oxidation level was reached at 0.3 sec from light activation) with respect to WT and mOE plants (where 0.7 and 1.2 sec were respectively needed to reach half of the maximal P700 oxidation, Figure 3a; Figure S6b), suggesting that an increased FLV pool enabled a larger PSI oxidation through their electron acceptor capacity.

**Figure 4:**
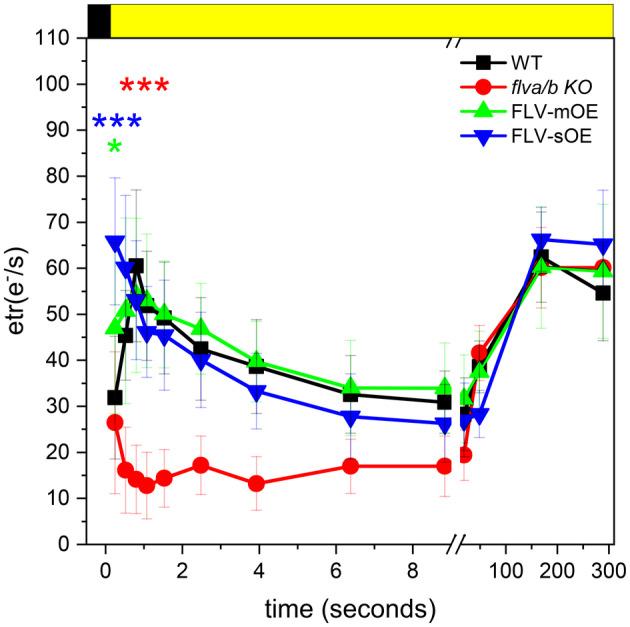
FLV contribution to the electron transport upon light activation. Ten‐days‐old protonema of WT, *flva/b* KO and 6 FLV‐OE lines are used to test ETR (e^−^/s) at the JTS‐10. Forty minutes dark‐adapted samples are exposed to 5 min of 300 μmol photons m^2^ sec^−1^ red light to activate photosynthesis. The detected ECS used to calculate the final ETR has been normalized to the PSI + PSII total amount (see “Materials and methods” section). Graph reports average ± SD of *n* > 4 biological replicates respectively for WT, *flva/b* KO, FLV‐mOE, and FLV‐sOE lines are shown in black, red, green, and blue respectively. Statistically significant differences between WT and mutant lines is indicated by asterisks with the color code of the correspondent lines (*t*‐test, *P = 0.05; ***P < 0.01).

The impact of different FLV accumulation levels was further assessed by measuring the effect on ETR capacity upon light activation, estimated from pmf generation across the thylakoids membrane by monitoring ECS signal with a DIRK approach (Sacksteder & Kramer, 2000). *P. patens* WT plants could activate a strong ETR already 0.5 sec after light is switched on, a capacity lost in *flva/b* KO (Figure 4) and thus associated with the presence of active FLV. This extra electron transport capacity was impactful for a few seconds under strong illumination while already after 20–30 sec WT and *flva/b* KO became indistinguishable. Indeed, at stationary state after 5 min of illumination, FLV absence did not have any significant impact on estimated ETR (Figure 4).

FLV‐mOE lines showed a complete rescue of the *flva/b* KO ETR phenotype with WT‐like electron transport kinetic (Figure 4). Interestingly, ETR capacity of FLV‐sOE resulted even larger than the WT at the onset of light activation (Figure 4; Figure S6c). In accordance with the faster light‐driven P700 oxidation kinetic (Figure 3; Figure S6a), ETR results suggested that the larger accumulation of active electron sinks in FLV‐sOE lines enhanced electron transport rate immediately after illumination. This difference was, however, only visible for a very short time. Already after 2 sec of illumination, both FLV‐mOE and FLV‐sOE lines ETR was indistinguishable from the WT one and resulted even overlapping with *flva/b* KO ETR at steady state. Indeed, after a few minutes of illumination. (e.g., minute 5 in Figure 4), when major energy‐consuming pathways such as CO_2_ fixation are fully activated, FLV contribution to ETR was not detectable anymore (Figure 4).

### FLV impact on PSI and PSII efficiency is not transient in the absence of a proton gradient

**Figure 5:**
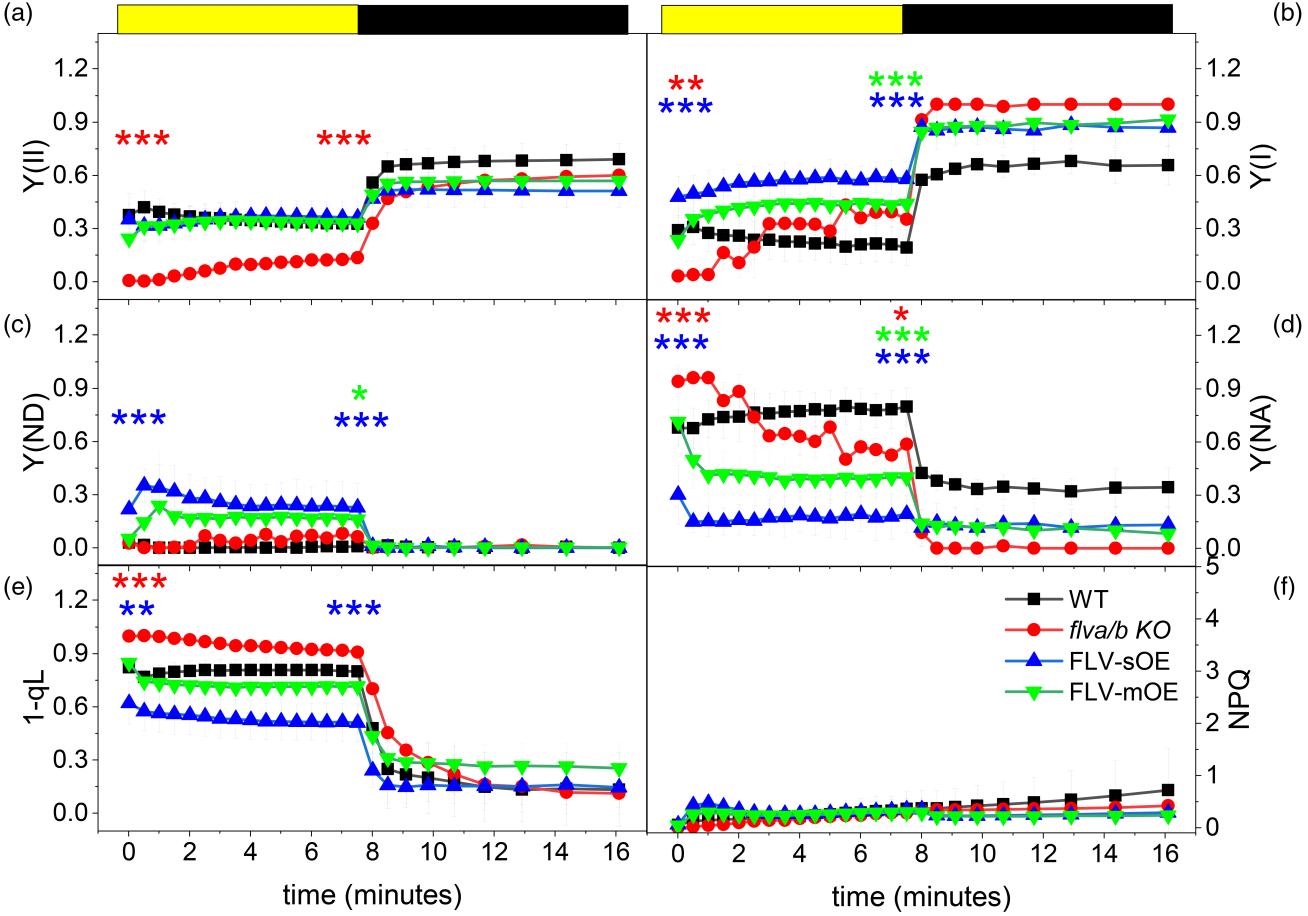
Impact of FLV overexpression on PSII and PSI activity in nigericin‐treated plants. *Physcomitrium patens* plants, dark‐adapted and nigericin treated for 40 min, were exposed for 8 min to 165 μmol photons m^2^ sec^−1^ light followed by 8 min of darkness. (a–f) Graphs report, respectively Y(PSII), Y(PSI), Y(ND), Y(NA), a fraction of closed reaction centers (1‐qL) and NPQ values. WT, *flva/b* KO, FLV‐mOE, and FLV‐sOE lines are shown respectively as black squares, red circles, green triangles, and blue triangles. FLV‐sOE and FLV‐mOE reports averaged data from three independent lines. Statistically significant differences between WT and mutant lines, at the beginning and end of light treatment, are indicated by asterisks with the color code of the correspondent lines (*t*‐test, *P = 0.05; **P = 0.01; ***P < 0.01).

The same measurements in Figure 2 were also repeated in the presence of nigericin, a protonophore able to dissipate light accumulated ΔpH and thus inhibiting NPQ activation and photosynthetic control (Finazzi et al., 2004; Takahashi et al., 2009; Tóth et al., 2020). Treatment was effective on *P. patens* tissues, as shown by the full suppression of NPQ in all tested lines (Figure 5f).

Untreated WT usually presented an elevated PSI donor side limitation when exposed to strong illumination (Figure 2; Figure S7c). Nigericin inhibits photosynthetic control and affects PSI yield. Most importantly PSI becomes limited from PSI acceptor side (Figure S7b,d). Nigericin‐treated *flva/b* KO *P. patens* exhibited even lower PSI and PSII efficiency, higher PSI acceptor side, and lower closed PSII reaction centers at the onset of light activation compared with treated WT plants (Figure 5a–e). Interestingly, *flva/b* KO plants took several minutes to recover and reach WT photosynthetic parameters.

All complemented lines incubated with nigericin, rescued PSII and PSI efficiency as well as Y(ND), Y(NA), and 1‐qL compared with *flva/b* KO recipient line at the onset of light activation (Figure 5a–e) thus suggesting how FLV were functional in the presence of the inhibitor. Moreover, FLV‐sOE showed higher Y(I), lower Y(NA), and 1‐qL with respect to WT plants, demonstrating a considerably improved ability to respond to illumination with respect to WT in the presence of the inhibitor.

Even more remarkable was the observation that in the presence of the inhibitor, the differences associated with FLV accumulation levels were stable for the whole illumination period with FLV‐sOE showing the highest Y(I), the lowest PSI acceptor side limitation (Figure 5b,d) and PSII saturation from the dark‐to‐light transition until the end of illumination treatment (Figure 5e). These data demonstrated that at least in the presence of the inhibitor, FLV activity is not necessarily transient since it impacted photosynthetic properties and it can be detected also at steady conditions.

### FLV overexpression limits *P. patens* growth

**Figure 6:**
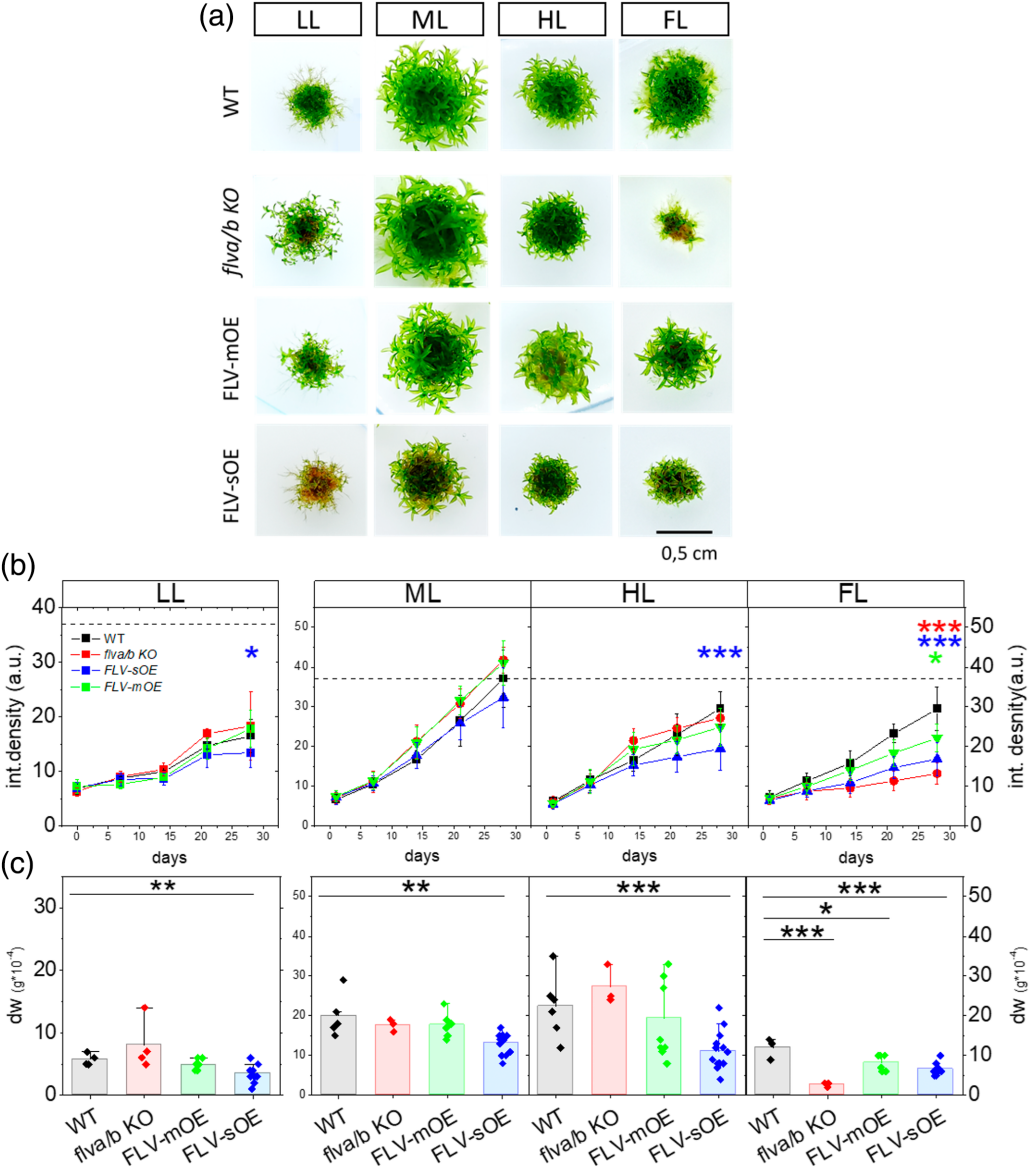
Growth phenotype under LL, ML, HL, and FL. (a) Representative pictures of 28‐days‐old WT, *flva/b KO*, FLV‐mOE, and FLV‐sOE grown under constant LL, ML, HL, and FL (the clones #3 and #12 have been used as representatives respectively of FL V‐mOE and FLV‐sOE). All plants were grown under a 16/8 h photoperiod. (b) Twenty‐eight days growth curve of WT, *flva/b* K/O, FLV‐mOE, and FLV‐sOE (respectively as black squares, red circles, green triangles, and blue triangles). Graphs report average ± SD of *n* > 4 replicates where FLV‐sOE and FLV‐mOE report averaged data from three independent lines. (c) Dry weight (expressed in g × 10^−4^) of 28‐days‐old WT, *flva/b* KO, FLV‐mOE and FLV‐sOE grown under (respectively in black, red, green, and blue) LL, ML, HL, and FL. Graphs report data of *n* > 4. Significant differences between WT and the correspondent lines are reported with asterisks keeping the color code of the line under investigation (*t*‐test, *P = 0.05; **P = 0.01; ***P < 0.01).

To assess the impact of FLV overaccumulation on growth, plants were incubated in different light regimes for 28 days: constant low (LL), medium (ML), high (HL), and fluctuating light (FL, Figure 5a), always with a 16/8 h photoperiod. WT, *flva/b* KO, and FLV‐mOE growth was undistinguishable under all constant illumination (LL, ML, and HL), independently if the light was limiting or in excess (Figure 6a,b). FLV presence was dispensable under constant light regimes and no differences were detected between WT and *flva/b* KO (Figure 6; Figure S7a). However, in all these conditions, FLV‐sOE showed a reduction both in size (Figure 6b) and biomass accumulation (Figure 6c) demonstrating a penalty associated with the high protein accumulation in conditions of steady illumination. In the case of HL, but not LL or ML, FLV‐sOE also showed a reduction in *F*_v_/*F*_m_ indicative of a possible photodamage (Figure S7b).

While FLV absence was not impactful in steady illumination conditions, *flva/b* KO showed a major phenotype penalty in FL condition (Figure 6, Storti, Puggioni, et al., 2020) associated with a lower *F*_v_/*F*_m_ indicative of a photodamage (Figure S7b). FLV reintegration indeed rescued the *flva/b* KO phenotype since both FLV‐sOE and FLV‐mOE exposed to FL showed improved growth with respect to the parental line, even though the rescue was not complete (Figure 6b,c), as confirmed also by the low *F*_v_/*F*_m_ (Figure S7b).

## DISCUSSION

### FLV overexpression rescues photosynthetic electron transport after a sudden increase in incident light but has potential impact to act steady state

In this work, *P. patens flva/b* KO plants were complemented with a construct expressing both FLVA and FLVB separated by a 2A peptide (Yamamoto et al., 2016). The approach was selected to achieve a similar expression level of the two isoforms, that are active as heterocomplex (Beraldo et al., 2024; Gerotto et al., 2016), and it indeed enabled to obtain plants with different levels of overexpression but always with comparable FLVA and B accumulation (Figure 1; Figure S3). Lines exhibiting varying levels of FLV accumulation were obtained (FLV‐mOE and sOE) likely because of the insertion of multiple copies of the expression cassette in the same locus, a quite common occurrence in *P. patens* (Kamisugi et al., 2006).

All overexpressing lines showed active FLV, as confirmed by the rescue of *flva/b* KO photosynthetic parameters during a dark‐to‐light transition (Figures 2, 3, and 4). The ability to keep PSI oxidized after a light increase is particularly physiologically relevant since PSI is sensitive to over‐reduction damage in case of acceptor side limitation (Tiwari et al., 2016). Indeed, *flva/b* KO shows high acceptor side limitation under fluctuating light exposure, and this corresponds to a strong growth penalty in these conditions (Figure 6; Figure S8). The partially rescued phenotype of FLV‐sOE and FLV‐mOE confirms the previous data showing that FLV are active in protecting PSI by acting as electron acceptor upon sudden increases of illumination (Figure 6; Figure S8; Allahverdiyeva et al., 2013; Gerotto et al., 2016).

In dark‐to‐light transition analyses, the plants with the largest protein accumulation (FLV‐sOE), showed a higher donor side limitation and lower acceptor side limitation (Figure 2) than the WT plants. This can be explained by improved photosynthetic control or by enhanced electron transport from PSI immediately after exposure to saturating light. In FLV‐sOE plants, Y(ND) and Y(NA) are still respectively high and low even in the presence of nigericin, that dissipates ΔpH and thus inhibits photosynthetic control (Figure 5). This supports the latter hypothesis confirming that FLV accumulation impact is due to its ability to accept electrons downstream of PSI, increasing electron transport capacity and thus enabling a faster P700 oxidation (Figures 3 and 4).

It should however be underlined that this additional electron transport capacity detected in FLV‐sOE plants is quite small, despite the much higher (>20 times) protein accumulation, indicating that the potential beneficial effects from FLV are already almost saturated in *P. patens* WT plants and that any additional protein accumulation has a limited impact. This is confirmed by the observation that many parameters, such as Y(II) upon light activation (Figure 2), maximal P700 oxidation capacity (Figure 3; Figure S6a) and Pm assessed upon exposure to 1 h light fluctuations (Figure S5), all were indistinguishable between WT and FLV‐OE. Overall, these analyses show that while the absence of FLV in *flva/b* KO has a clear impact on the ability to respond to light fluctuations, the increased accumulation does not provide an equivalent improvement.

All genotypes, including *flva/b* KO, have indistinguishable steady‐state ETR and fluorescence parameters suggesting that FLV impact is absent or at least not detectable anymore once CO_2_ fixation is fully activated. Interestingly, this was not the case for nigericin‐treated plants where the impact of FLV overaccumulation was detectable also at a steady state (Figure 5; Figure S7). Usually, in WT plants, PSI is limited from the acceptor side only in the dark‐to‐light transition, and in these conditions, FLV activity is detectable. If photosynthetic control is inhibited, PSI remains limited from the acceptor side also at steady‐state and FLV activity becomes detectable (Figure 5). Nigericin treatment thus makes indispensable FLV activity also at steady illumination with FLV‐overexpression significantly reducing Y(NA) at steady‐state compared with the *flva/b* KO, WT, and FLV‐mOE lines (Figure 5).

These results on one side demonstrate that photosynthetic control is essential for ETR regulation. If photosynthetic control is inhibited, a higher accumulation of FLV, not normally impactful, becomes effective in reducing PSI over‐reduction (Figure 5). On the contrary, if photosynthetic control is active in reducing electron flux from Cyt b_6_f additional FLV activity is undetectable.

On the other side, these data also show that at least when *P. patens* plants are treated with a protonophore FLV activity can be detected at a steady state. FLV activity is thus not necessarily transient, but this happens because there are no extra electrons available at the PSI acceptor side once carbon fixation and photosynthetic control are active. This observation opens the possibility that even if its impact is not visible comparing steady‐state ETR and fluorescence parameters in WT and *flva/b* KO plants, some FLV activity at steady state could be present but be undetectable. In fact, it is well known that different mechanisms acting as alternative electron transport pathways *in vivo* have a strong complementarity (Munekage et al., 2004; Storti et al., 2019; Yamamoto et al., 2016) and thus the loss of a small constitutive FLV contribution in *flva/b* KO could be undetectable in mutants because it is compensated by a corresponding increase of cyclic electron transport.

Indeed, there is a few evidence in the literature consistent with the possibility that some FLV activity could be present even at steady‐state. Prolonged analyses of P700 redox kinetic (up to 50 sec; Helman et al., 2003) of *flv1/3 KO* lines showed that FLV acts as an electron sink for a prolonged time, keeping P700 oxidized at a steady state in cells where RuBisCO was chemically inhibited. Another example comes from *P. patens pgrl1/ndhm* KO depleted in cyclic electron transport. While these plants grow well in steady mild illumination, if FLV is also depleted the resulting *pgrl1/ndhm/flva* KO plants show a major growth reduction in all conditions. This suggests that, at least in a *pgrl1/ndhm* KO mutant context, FLV are capable of sustaining a basal activity under constant illumination even though this is not detectable comparing only *flva* KO and WT plants (Storti, Segalla, et al., 2020).

While these examples all come from organisms where ETR was altered by mutations and inhibitors treatments and thus may not be fully representative of the state of WT plants, these results demonstrate that constitutive FLV activity is possible and suggest that its contribution may have been underestimated in analyses of simple mutants.

### FLV overaccumulation can cause growth penalties

**Figure 7:**
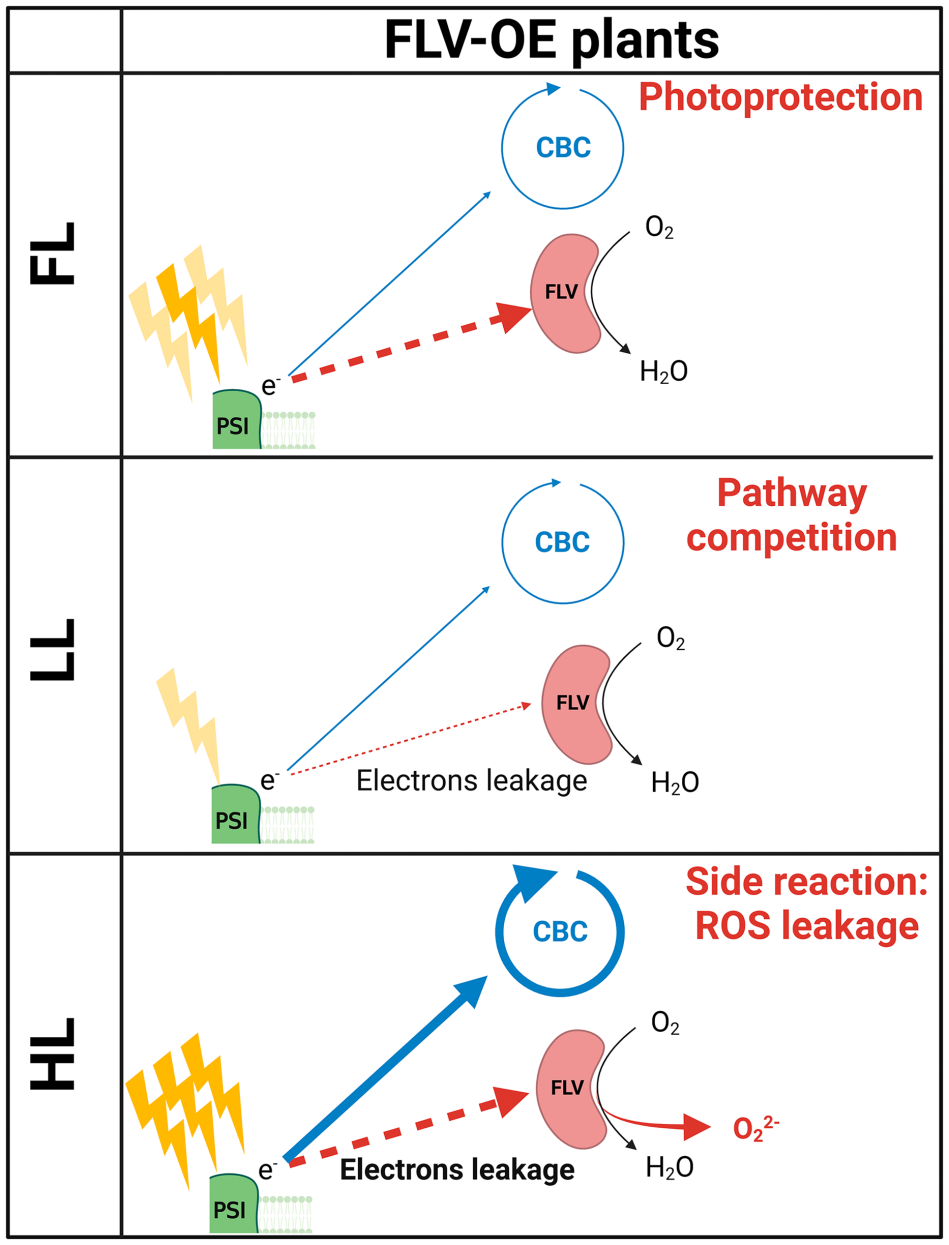
Representation of electron paths in FLV‐OE lines. Schematic representation of PSI‐electrons destiny under different light conditions in FLV‐sOE plants. Under FL, the larger FLV pool can anyway act as photoprotective mechanism. Under LL, electrons leaking to the larger FLV pool induces competition with CBC. Under HL, electrons leaking to the larger FLV pool increases and FLV ROS‐producing side reaction becomes significant.

FLV were lost during the evolution of angiosperms where their protective activity was suggested to be at least partially compensated by increased cyclic electron transport (Gerotto et al., 2016). Recent experiments showed that FLV are active if expressed in Arabidopsis and rice (Basso et al., 2022; Wada et al., 2018; Yamamoto et al., 2016) where they compensate for the inactivation of cyclic electron transport without any measurable competition with CO_2_ fixation (Basso et al., 2022; Wada et al., 2018). Furthermore, FLV expression in angiosperms showed positive effects in Arabidopsis plants exposed to drought stress (Tula et al., 2020) and reduced ROS production in tobacco plants under drought and salt stresses (Vicino et al., 2021). *P. patens* FLV‐OE results are well consistent with literature data in different organisms (Bag et al., 2023; Basso et al., 2022; Tula et al., 2020; Vicino et al., 2021) showing how FLV activity is beneficial in response to FL. Indeed, FLV over‐accumulating plants maintained their ability to accept electrons from PSI (Figure 2; Figures S3 and S4) and to use them for O_2_ reduction into water, thus avoiding over‐reduction and consequent PSI photodamage under FL (Figure 7).

These positive results are puzzling considering that FLV were lost during the evolution of angiosperms, which would imply the existence of some negative impact associated with their presence. Since FLV is currently present in many organisms, cyanobacteria, eukaryotic algae, non‐vascular plants, and gymnosperms (Ilík et al., 2017), its activity cannot simply be detrimental in present CO_2_, Oxygen and light conditions currently experienced by plants or we would expect natural selection to have driven its loss during evolution of these other organisms as well. Therefore, there must have been a phase in angiosperms evolution or in their distinct developmental features where FLV become negative or at least superfluous.

The negative effects observed on growth, in different light regimes, due to FLV overaccumulation (Figure 6) can provide unique insights to address this question and propose a reasonable hypothesis. It is particularly intriguing to observe that OE plants show a growth penalty under constant illumination, especially under limiting (LL) and strong (HL) illumination (Figure 6). These negative effects are also correlated with the protein accumulation, being stronger in FLV‐sOE than in the lines with lower accumulation confirming this is associated with protein activity.

To explain this phenotype in association with FLV activity in LL and HL, two requirements are needed: (i) FLV must be active constitutively; (ii) their activity must represent a penalty. The former, as previously discussed, is a realistic possibility, as shown in CBC‐impaired lines (Helman et al., 2003), CEF depleted plants (Storti, Puggioni, et al., 2020) and by the impact on photosynthetic parameters measured in nigericin‐treated FLV‐sOE at steady‐state (Figure 5). If this first assumption is realistic, the growth penalty in plants exposed to constant LL could be explained by the energy waste due to electrons leaking through FLV‐pathway rather than being funneled to CBC and the rest of metabolism (Figure 7).

It is also possible that the strong protein overexpression causes a high metabolic cost detrimental in limiting light. This hypothesis, however, could explain why the OE plants showed growth reduction also in excess illumination, where more energy waste would be beneficial. On the contrary, FLV‐sOE plants show a reduced growth in HL as well, while both FLV‐mOE and *flva/b* KO were indistinguishable from WT (Figure 6), demonstrating a specific negative effect associated with FLV overaccumulation. Under HL this growth penalty is likely associated with some photodamage, as shown by the severe *F*_v_/*F*_m_ decrease with increasing FLV accumulation level (Figure S8b). A mechanistic hypothesis to explain the negative impact of FLV overaccumulation can be found analyzing available information on the reaction mechanism of FDP, the homologous proteins found in non‐photosynthetic organisms with the same catalytic activity. If FLV in photosynthetic organisms has a conserved reaction mechanism, as expected, it will follow a two‐step reaction (Frederick et al., 2015), where a fully reduced FLV reacts with oxygen generating a peroxo‐diferric group that is rapidly reduced by the proximal FMNH_2_ to produce water. According to this hypothesis, when the enzyme is working efficiently, the intermediates are readily consumed and water is safely produced from oxygen. It is however possible that some side reactions can occur with the formation of spurious intermediate ferryl species that drive to ROS formation (Gray & Winkler, 2021; Martins et al., 2019). While this should normally be an uncommon occurrence, it is possible that FLV has a small ROS leakage because of the incomplete O_2_ reduction that becomes impactful in plants with strong overaccumulation under excess illumination (Figure 7).

Interestingly, a similar hypothesis has been suggested for plastid terminal oxidase (PTOX) overexpression in *Arabidopsis thaliana* and *Nicotiana tabacum* (Heyno et al., 2009). PTOX is a plastoquinol oxidoreductase carrying a non‐heme di‐iron center in its active site and active in keeping the electron transport chain oxidized reducing O_2_ into H_2_O. While its overexpression was advantageous for plants exposed to LL, it unexpectedly increased ROS production under HL. This outcome was suggested to be linked to ROS‐producing side reactions when the substrate availability was not sufficient to quickly complete O_2_ reduction (Heyno et al., 2009).

These observations suggest that FLV, while they are advantageous under light fluctuations, their accumulation causes a growth penalty under steady illumination conditions. In a natural dynamic environment, both contributions are expected to be present, generating a tradeoff. It is however conceivable an evolutionary path where during a phase of their evolution, angiosperms' ancestors encountered conditions where the growth penalty overcome the advantages, driving to the evolutionary loss of FLV. This would have been made possible also because other mechanisms, such as CET and photorespiration, increased their capacity in angiosperms (Rizzetto et al., 2025; Smith et al., 2024; Storti et al., 2019) and were able to at least partially compensate for the loss of FLV electron transport upon a sudden light increase.

## MATERIALS AND METHODS

### Generation of plant material

To generate the *flva/b* double knock‐out mutant (*flva/b* KO), the FLVB locus was disrupted from the genome of *flva* KO plants by homologous recombination (Gerotto et al., 2016), using the same constructs previously adopted (Gerotto et al., 2016) for the successful isolation of single *flvb* knock‐out strains. Transformants were screened by PCRs from genomic DNA (gDNA) and gene disruption was validated by RT‐PCRs from cDNA with specific primers (Figure S1a). To generate FLV Over‐Expressing lines (FLV‐OE), *flva/b* double KO plant #2 (Figure S1a) has been transformed with FLVA‐2A‐FLVB (kindly provided by Prof. Shikanai; Yamamoto et al., 2016) cloned into PT1OG plasmid (#LC126301.1, NCBI GenBank), where its expression is driven by the EF1‐α strong promoter.

### Growth conditions and treatment

Protonemal tissue of *P. patens* of Gransden wild‐type (WT), *flva/b* KO and the isolated FLV‐mOE and FLVs‐OE lines were grown on minimum PpNO_3_ media in controlled conditions: 21°C, 16 h light/8 h dark on photoperiod and light intensity of 50 μmol photons m^−2^ sec^−1^ (control light, CL), and analyzed after 10 days of growth. The growth phenotype of WT, *flva/b* KO, FLV‐mOE and FLV‐sOE lines was evaluated by growing them at different light conditions at 21°C, 16 h light/8 h dark: constant 25 μmol photons m^−2^ sec^−1^ (low light, LL), 50 μmol photons m^−2^ sec^−1^ (medium light, ML), 300 μmol photons m^−2^ sec^−1^ (high light, HL), and cycles of 1 min at 800 μmol photons m^−2^ sec^−1^ followed by 5 min at 25 μmol photons m^−2^ sec^−1^ (fluctuating light, FL) and homogeneous circles of 2 mm diameter on solid medium were the growth test starting material. Spots dimension was monitored by scanning the whole plates at different time points over 28 days and calculated as integrated density (area × mean intensity) as reported in Storti et al. (2019). The 28‐days‐old spots were dried for 24 h at 60°C to evaluate the dry weight.

### Total protein extract, SDS‐PAGE, and immunoblotting

Total extracts were obtained by grinding tissues in solubilization buffer [50 mm TRIS (VWR‐Italy, #0497) pH 6.8, 100 mm DTT (Thermo Fisher Scientific‐Italy, #R0862), 2% SDS (Sigma‐Italy, #62862), and 10% glycerol (Sigma‐Italy, #G9012)]. For immunoblotting analysis, following SDS‐PAGE, samples were loaded at the same chlorophyll content. Proteins were transferred to nitrocellulose membranes (Millipore, Sigma‐Italy #HATF09025) and detected with both Horseradish Peroxidase (HRP, Agrisera‐Sweden; #AS09‐60s) or Alkaline Phosphatase‐conjugated secondary antibody (Sigma‐Italy; #A3562) after hybridization with specific primary antibodies (anti‐PsaD, Agrisera‐Sweden; #AS09 461; anti‐Cyt f; Agrisera‐Sweden; #AS06 119; custom‐made anti‐FLVA, and anti‐FLVB (Gerotto et al., 2016); and anti‐D2, in house polyclonal antibodies). Anti‐PsaD recognizes a double band, likely corresponding to two protein isoforms as identified by mass spectrometry (Busch et al., 2013). CHEMI premium imager (VWR‐Italy) was used for HRP‐conjugated protein detection.

### Spectroscopic analyses

Chlorophyll fluorescence emission has been detected with FluorCAM 800MF from Photon System Instruments (PSI‐Drásov, Czechia). FLVs‐OE primary resistant clones were exposed to 5 min 150 μmol photons m^−2^ sec^−1^ non‐saturating light. Values of *F*_v_/*F*_m_ and $F_{v}^{\prime }/F_{m}^{\prime }$ calculated respectively as (*F*_m_ − *F*_o_)/*F*_m_ and $F_{m}^{\prime }-F_{o}^{\prime }/F_{m}^{\prime }$.

Fluorescence and P700 measurements were carried out with Dual‐PAM‐100 fluorometer (Walz‐Germany) on 10‐day‐old plants grown on PpNO_3_ under control light (50 μmol photons m^−2^ sec^−1^). Prior to the analyses, plants were dark acclimated for 40 min and placed onto glass fiber filters (Millipore, Sigma‐Italy #APFC02500). For induction‐recovery kinetics samples (with and without nigericin, 35 mg μl^−1^), actinic constant red light was set at 165 or 500 μmol photons m^−2^ sec^−1^ for 8 min and switched off for the following 8 min. For Pm evaluation after fluctuating light response, cycles of 1 min 800 μmol photons m^−2^ sec^−1^‐4 min darkness are repeated for 1 h. PSII and PSI‐related parameters were calculated upon saturating pulses (6000 μmol photons m^−2^ sec^−1^, 600 msec) as follows: Y(II), $F_{m}^{\prime }-F_{o}/F_{m}^{\prime }$; Y(I), 1‐Y(ND)‐Y(NA); Y(NA), (Pm‐Pm′)/Pm; Y(ND), (P‐Po/Pm) (Klughammer & Schreiber, 1994). For the fast‐kinetic, samples [with and without 3‐(3,4‐dichlorophenyl)‐1,1‐dimethylurea, DCMU 40 μm] have been exposed to 6 sec of saturating high light (20 000 μmol photons m^−2^ sec^−1^) and 6 sec of darkness and P700 oxidation signals have been recorded in real‐time. To evaluate the total oxidable P700 (P700^+^) it has been used the repetitive Saturating Pulse (rSP) protocol (Shimakawa et al., 2018): 40 min dark‐adapted plants exposed to six 1‐sec‐SPs (2000 μmol photons m^−2^ sec^−1^) repeated every 10 sec (total exposure time of 1 min) under a constant light (75 μmol photons m^−2^ sec^−1^). The total oxidable P700 was evaluated during the last 1 sec SP and compared with the total P700 content (totP700) assessed on dark‐adapted plants exposed to 10 sec Far Red (FR) light and lightened with 6000 μmol photons m^−2^ sec^−1^ for 600 msec.

Spectroscopic analysis was performed using a Joliot‐Type Spectrometer (JTS)‐10 spectrophotometer (Biologic) on 10‐day‐old plants grown in CL and kept wet in buffer‐infiltrated plants (Hepes 20 mm pH 7.5, KCl 10 mm). Electron transport rate (ETR) was evaluated by measuring the Electrochromic Shift (ECS) spectral change on plants exposed to 5 min of continuous light (300 μmol photons m^−2^ sec^−1^, 630 nm LED) after 40 min of dark adaptation (Gerotto et al., 2016). During this time, the light was rapidly switched off to calculate non‐photosynthetic contribution to ECS and the ETR was calculated as the ECS slope in the light subtracted by the slope calculated in the dark (Bailleul et al., 2010). The relative amount of functional PSI + PSII was evaluated by xenon‐induced single flash turnover and used to normalize electron transport rate values.

### ACCESSION NUMBERS

*Physcomitrium patens flva*: NCBI Gene ID 112291487; *Physcomitrium patens flvb*: NCBI Gene ID 112284006; Synthetic construct PpFlvA‐2A‐PpFlvB fusion protein gene, complete cds: NCBI GeneBank KT861472.1.

## AUTHOR CONTRIBUTIONS

AA and TM planned and designed the research; ET, CB, and MA performed experiments; ET, CB and AA analyzed data; ET, AA, and TM wrote the manuscript.

## CONFLICT OF INTEREST

The authors declare no conflicts of interest.

## Supporting information


**Figure S1.** *flva*, *flvb* and *flva/b* KO clones' characterization.
**Figure S2.** Screening of putative complemented lines.
**Figure S3.** Impact of FLV overexpression on PSI and PSII activity under strong illumination.
**Figure S4.** Impact of FLV overexpression on Y(NA) and Y(ND) non‐saturating light.
**Figure S5.** PSI photodamage under fluctuating light exposure.
**Figure S6.** ETR and P700 activation kinetic in the dark‐to‐light transition.
**Figure S7.** Effect of nigericin treatment on PSI and PSII efficiency of WT plant.
**Figure S8.** 28‐Days‐growth curve and *F*_v_/*F*_m_ of 7‐days‐old plants.
**Figure S9.** Model of FLV reaction mechanism and ROS leakage.

## Data Availability

Original data presented are deposited in public database: https://researchdata.cab.unipd.it/id/eprint/1474.
