# Supplementary material for: Flavodiiron proteins in Physcomitrium patens: navigating the edge between photoprotection and efficiency
Source: Plant J. 2025 Feb 24;121(4):e70052. doi: 10.1111/tpj.70052 (PMC11850960; doi:10.1111/tpj.70052)
Supplement: Supplementary file 1 — Figure S1. flva, flvb and flva/b KO clones' characterization. Figure S2. Screening of putative complemented lines. Figure S3. Impact of FLV overexpression on PSI and PSII activity under strong illumination. Figure S4. Impact of FLV overexpression on Y(NA) and Y(ND) non‐saturating light. Figure S5. PSI photodamage under fluctuating light exposure. Figure S6. ETR and P700 activation kinetic in the dark‐to‐light transition. Figure S7. Effect of nigericin treatment on PSI and PSII efficiency of WT plant. Figure S8. 28‐Days‐growth curve and F v/F m of 7‐days‐old plants. Figure S9. Model of FLV reaction mechanism and ROS leakage. [file TPJ-121-0-s001.pdf]

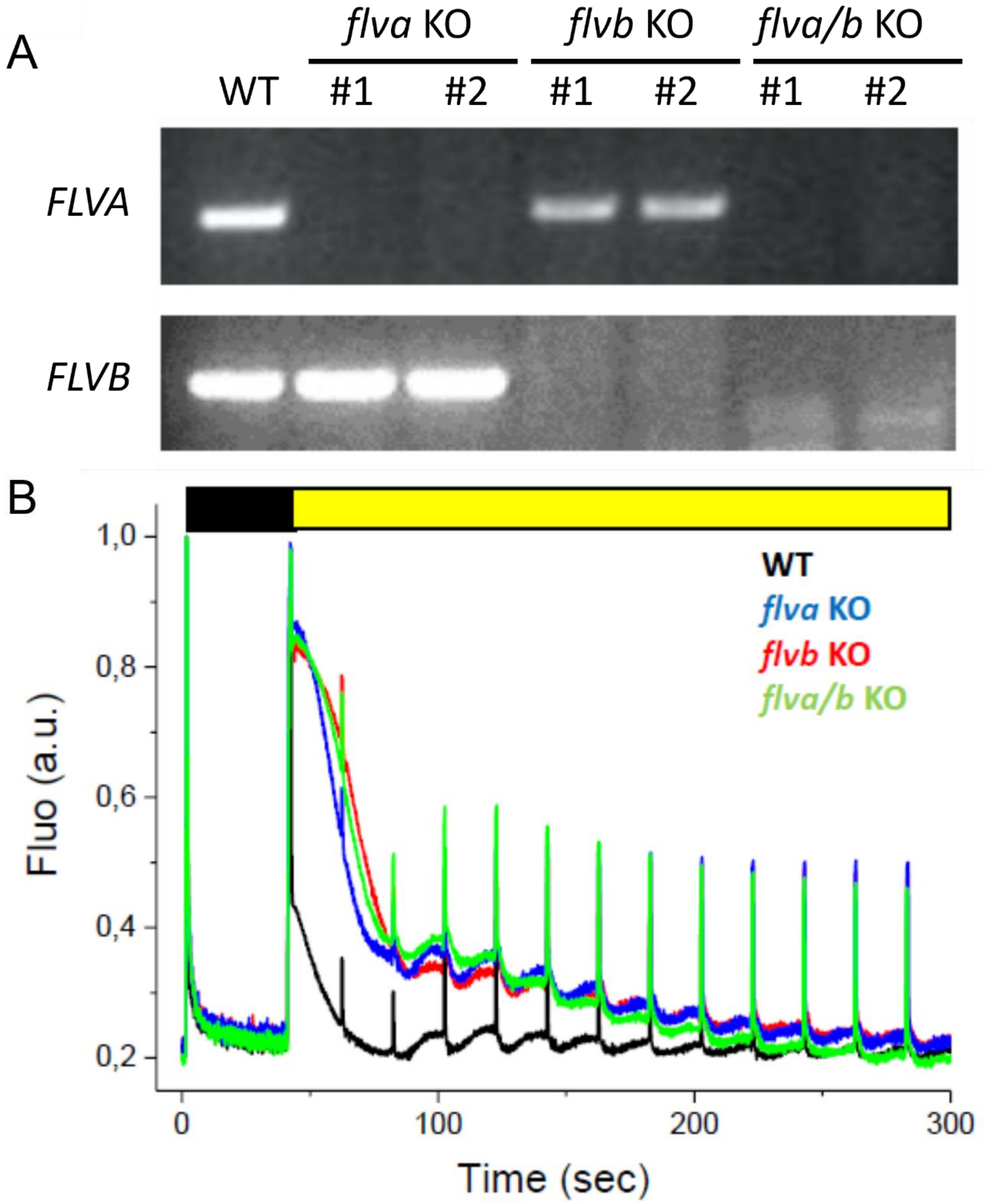

Figure S1. ***flva, flvb*** **and** ***flva/b KO*** **clones' characterization.**

A. *FLVA* and *FLVB* transcript accumulation in the different *flv* KO genotypes, showing that both FLVA and FLVB transcripts are missing in the double *flva/b* KO. #2-*flva/b* KO clone been used for FLVs-OE isolation. **B.** Chl fluorescence quenching in WT (black), *flva* KO (blue), *flvb* KO (red) and in the *flva/b* KO (green). *flva/b* KO show the same Chl fluorescence kinetics as *flva* and *flvb* single KO at the onset of light.

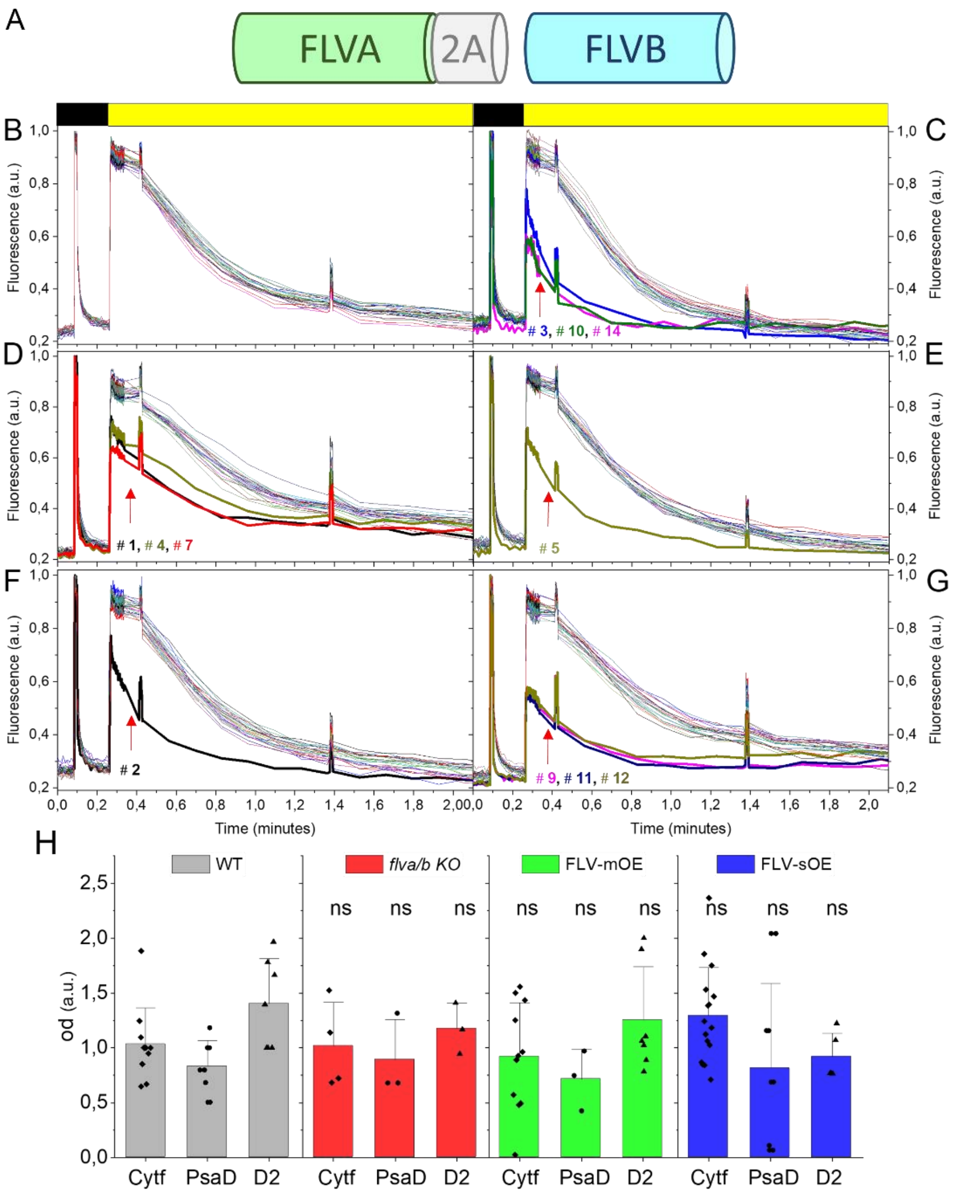

Figure S2. Screening of putative complemented lines.

A. *flva/b KO* line was transformed with FLVA-2A-FLVB construct, under the control of a constitutive promoter (EF1- α), coding for FLVA fused to 2A-peptide at the C-terminus and for FLVB. **B. C. D. E. F. G.** Chlorophyll fluorescence quenching kinetics of intact *P. patens* plants treated with 150 µmol photons m^{-2} s^{-1} actinic white light, as shown in Figure S1B. Each panel corresponds to an independent petri dish containing 25 independent lines. Red arrows point to lines with fast chlorophyll quenching, therefore putatively complemented for FLV activity and considered for further analyses. Panel A shows 25 lines with no putative complementation. **H.** Relative protein content of Cytf, PsaD and D2 was quantified by densitometry of western blotting. In all cases band intensity was normalized to the signal from WT plants in the same blot. WT, flva/b KO, FLV-mOE and FLV-sOE are represented respectively in black, red, green and blue.

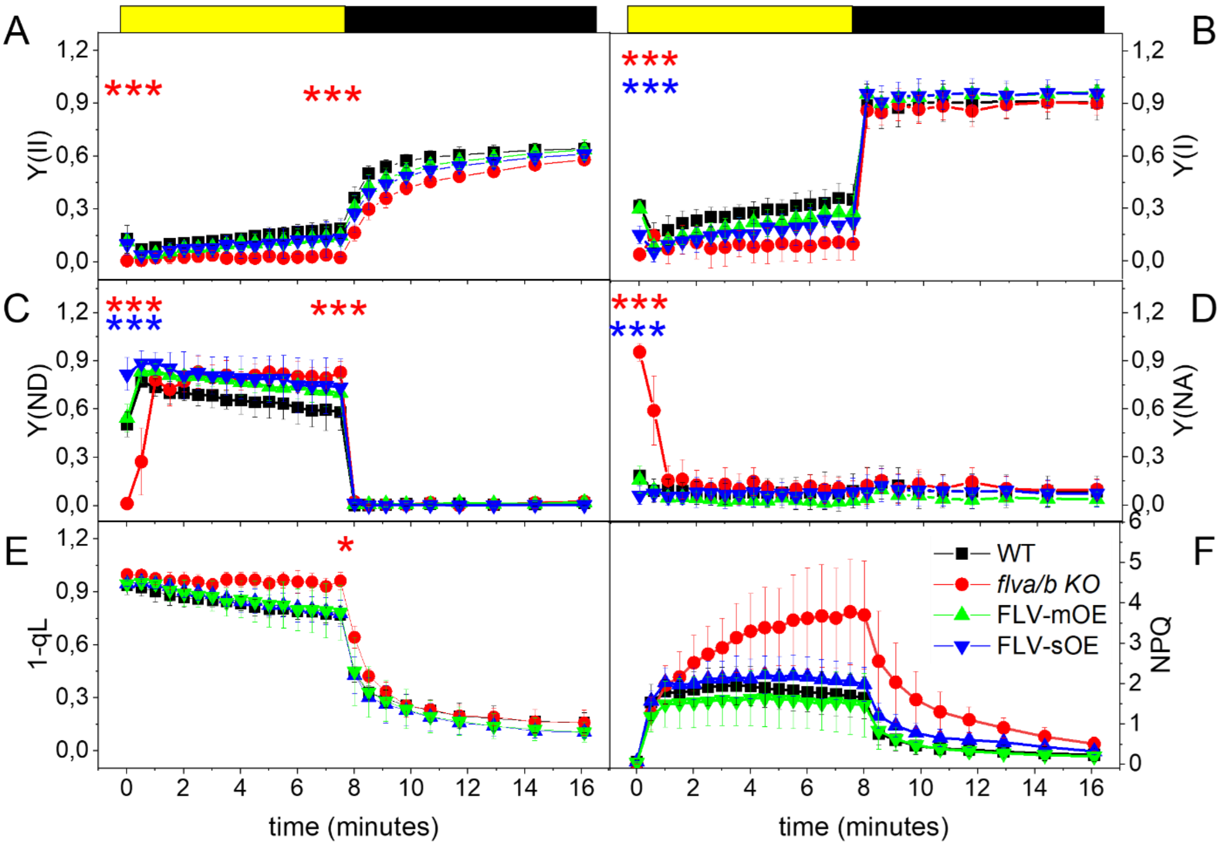

Figure S3. Impact of FLV overexpression on PSI and PSII activity under strong illumination.

The analysis shown in Figure 2 were also performed with stronger illumination. Dark adapted *P. patens* plants were exposed for 8 minutes to 500 µmol photons m^2 s^{-1} light followed by 8 minutes of darkness. Graphs **A, B, C, D, E** and **F** report respectively Y(PSII), Y(PSI), Y(ND), Y(NA), fraction of closed reaction centers (1-qL) and NPQ of WT, *flva/b* KO and FLV-mOE and FLV-sOE lines, shown respectively as black squares, red circles, green triangles and blue triangles. Statistically significant differences between WT and mutant lines, at the beginning and end of light treatment, is indicated by asterisks with the color code of the correspondent lines (t-test, *P = 0.05; **P = 0.01;***P<0.01)

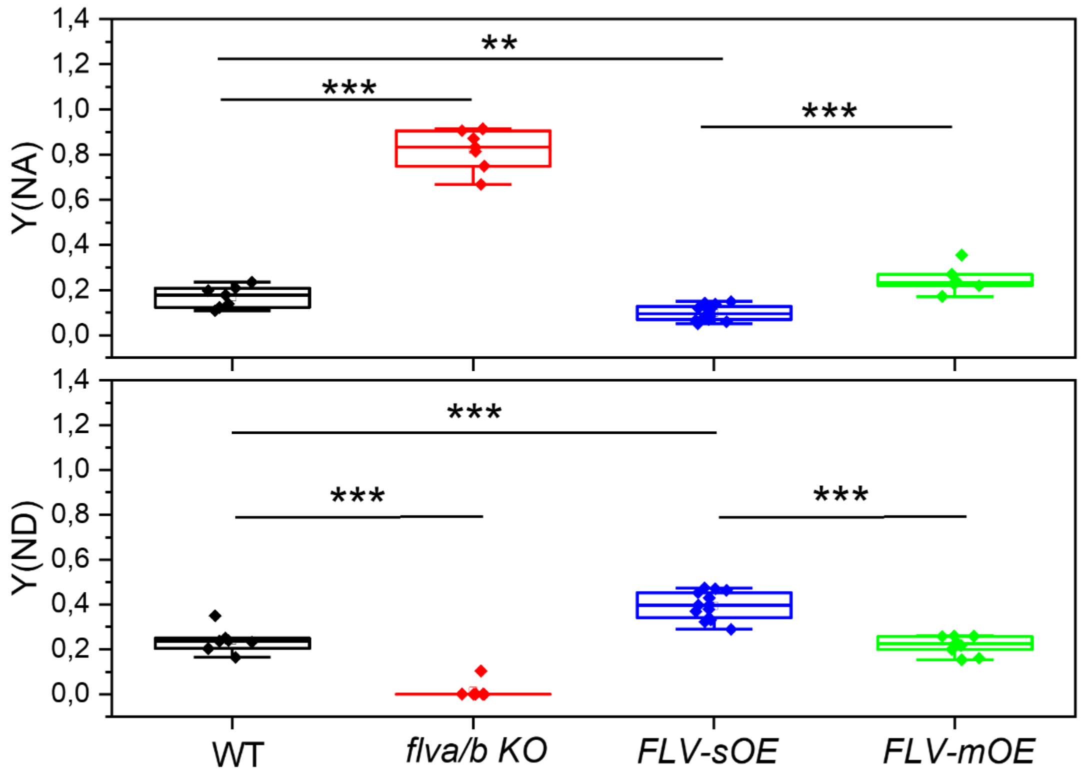

Figure S4. Impact of FLV overexpression on Y(NA) and Y(ND) non saturating light.

Dark adapted *P. patens* plants were exposed for 8 minutes to 165 µmol photons m^2 s^{-1} light followed by 8 minutes of darkness as reported in Figure 2. Graph reports PSI acceptor side, Y(NA) (**A**) and donor side Y(ND) (**B**) 1 second after light activation. WT, *flva/b* KO and FLV-sOE and FLV-mOE lines are shown respectively as black squares, red circles, blue triangles and green triangles. Average ± SD (n > 4) is shown. FLV-sOE and FLV-mOE like report average data from 3 independent lines. Asterisks indicate significant differences between WT and the correspondent lines (t-test, *P = 0.05; **P = 0.01;***P<0.01).

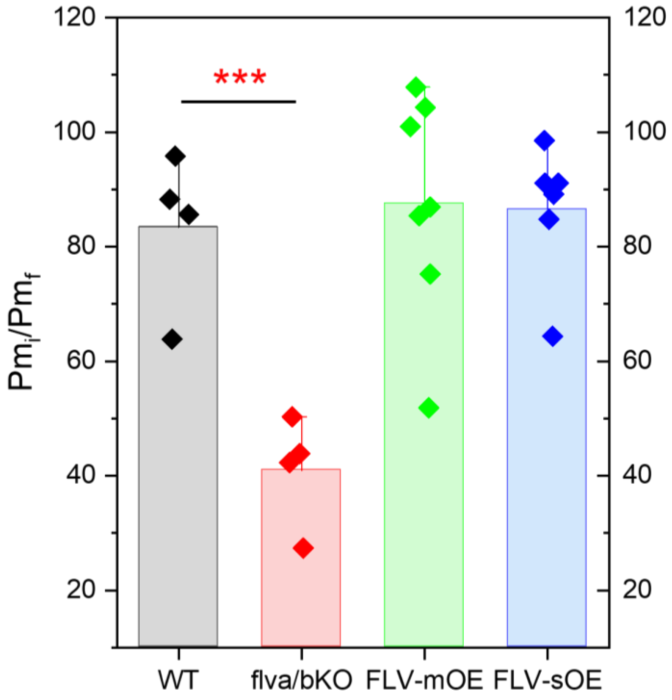

Figure S5. PSI photodamage under fluctuating light exposure.

Dark adapted *P. patens* plants were exposed for 1 hour to a fluctuating light protocol (1 minute at 800 µmol photons m^2 s^{-1} light followed by 4 minutes of darkness). Graph reports the ratio between Pm_i and Pm_f (Pm values recorded respectively at the beginning, Pm_i, and at the end, Pm_f, of the kinetic). Graph reports data of n > 4 biological replicates. FLV-sOE and FLV-mOE report data from 3 independent lines. Asterisks indicate significant differences between WT and the correspondent lines (t-test, *P = 0.05; **P = 0.01;***P<0.01).

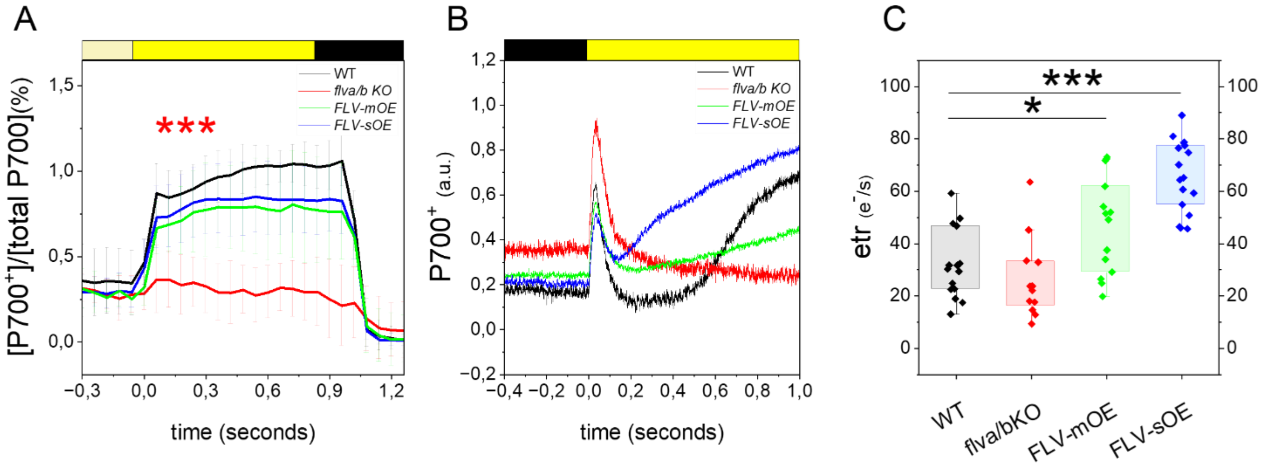

Figure S6. ETR and P700 activation kinetic in the dark-to-light transition.

A. P700 redox kinetic of the last 1-second-SP (2000µmol photons m^2 s^{-1}) after 1minute low light exposure (75 µmol photons m^2 s^{-1}) and 5 SPs (one every 10 seconds). $P700^+$ data have been normalized on Pm (tot P700). **B.** Magnification in the first second P700 redox kinetic reported in Figure 3A. **C.** First detected ETR value of the kinetic reported in Figure 4. WT, *flva/b* KO and 6 FLV-OE lines are used to test ETR (e^-/s) at the JTS-10. 40 minutes dark adapted samples are exposed to 5 minutes of 300 µmol photons m^2 s^{-1} saturating red light to activate photosynthesis. The detected ECS used to calculate the final ETR has been normalized to the PSI+PSII total amount (see material and methods). Graph reports data of n > 4 biological replicates with FLV-sOE and FLV-mOE report data from 3 independent lines. WT, *flva/b KO*, FLV-mOE and FLV-sOE have been reported in black, red, green and blue respectively. Asterisks indicate significant differences between WT and the correspondent lines (t-test, *P = 0.05; **P = 0.01;***P<0.01).

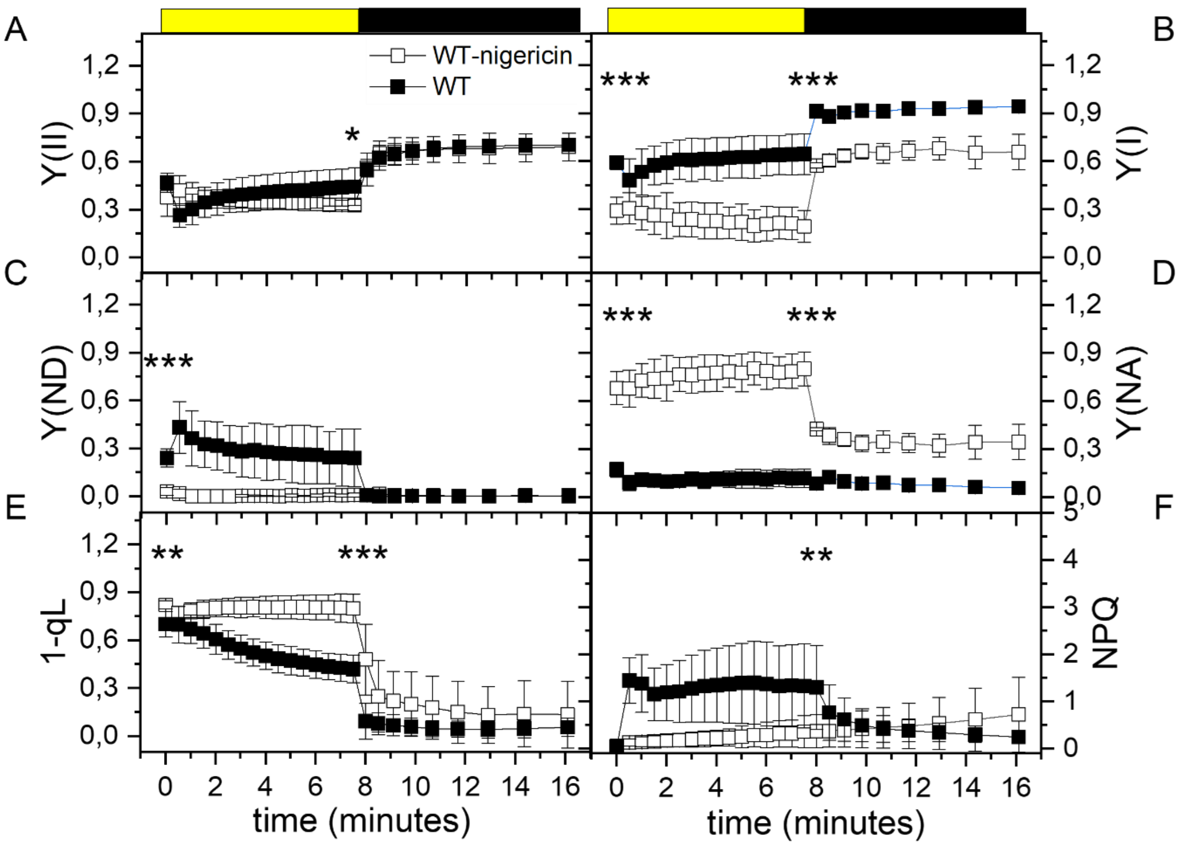

Figure S7. Effect of nigericin treatment on PSI and PSII efficiency of WT plant.

WT *P. patens* plants, dark adapted and either treated or not with nigericin for 40 minutes, were exposed for 8 minutes to 165 µmol photons m^2 s^{-1} light followed by 8 minutes of darkness. **A**. **B. C. D. E.** and **F.** graphs report respectively Y(PSII), Y(PSI), Y(ND), Y(NA), fraction of closed reaction centers (1-qL) and NPQ values. Untreated and treated WT lines data are shown respectively as solid or empty black squares. Average ± SD (n > 4) is shown. FLV-sOE and FLV-mOE report averaged data from 3 independent lines. Significant differences between WT and nigericin-treated WT, at the first and last point of light treatment, are reported with asterisks (t-test, *P = 0.05; **P = 0.01;***P<0.01).

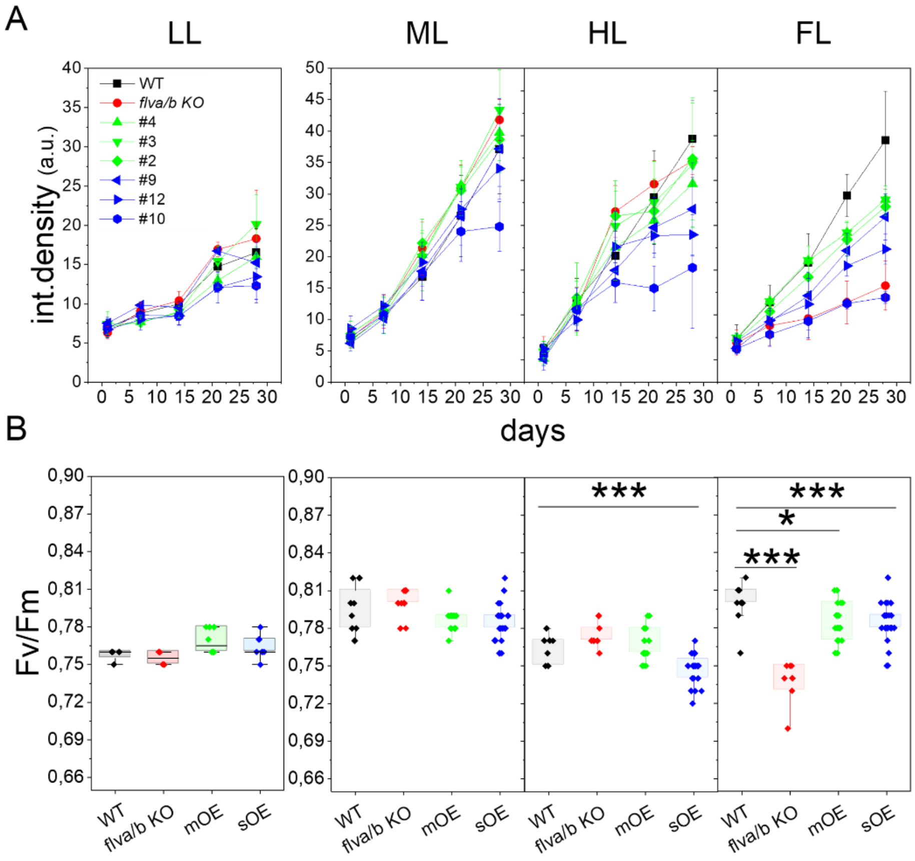

Figure S8. 28-days-growth curve and Fv/Fm of 7-days-old plants.

A. 28 days growth curve of WT, *flva/b* KO, FLV-mOE (#2, 3, 4) and FLV-sOE (#9, 10, 12) (respectively as black squares, red circles, green and blue shapes) grown under LL, ML, HL and FL respectively in the first, second, third and forth panels. **B.** Fv/Fm of 7 days old plants grown under LL, ML, HL and FL respectively in the first, second, third and fourth panels. Data of WT, *flva/b KO*, FLV-mOE and FLV-sOE have been reported in black, red, green and blue respectively. Graph reports data of n > 4 biological replicates. FLV-sOE and FLV-mOE report data from 3 independent lines. Significant differences between WT and the correspondent lines are indicated with asterisks (t-test, *P = 0.05; **P = 0.01;***P<0.01).

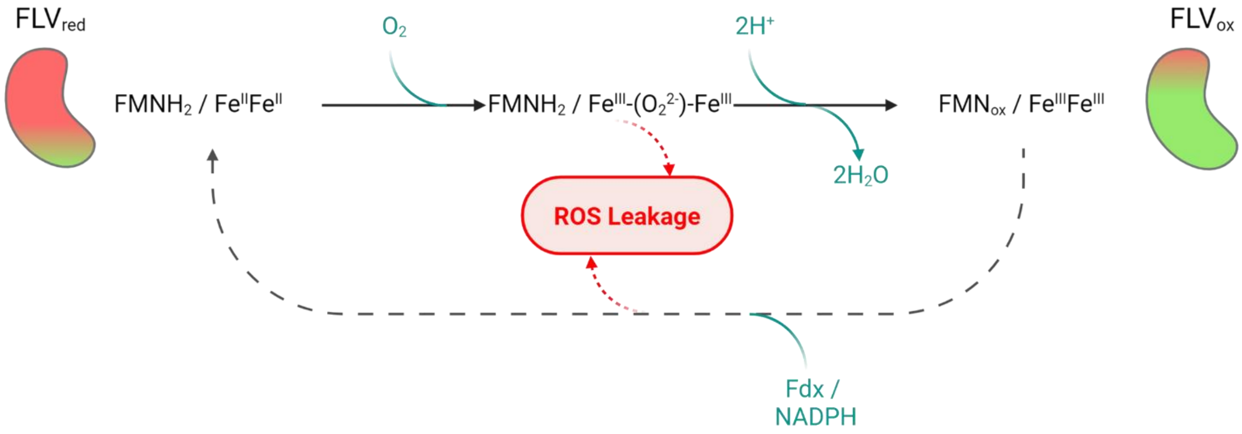

Figure S9. Model of FLV reaction mechanism and ROS leakage.

Proposed FLV reaction mechanisms, modified from (Frederick *et al.*, 2015). Fully reduced FLV (red) reacts with oxygen to generate water, oxidising two Iron atoms from II to III and $FMNH_2$ to FMN. Oxidised FLV (green) is re-reduced using four electrons transferred from Ferredoxin (Fdx) and possibly also NADPH.
